# Sleep loss causes social withdrawal and loneliness

**DOI:** 10.1038/s41467-018-05377-0

**Published:** 2018-08-14

**Authors:** Eti Ben Simon, Matthew P. Walker

**Affiliations:** 1 Center for Human Sleep Science, Department of Psychology, University of California, Berkeley, CA 94720-1650 USA; 2 Helen Wills Neuroscience Institute, University of California, Berkeley, CA 94720-1650 USA

## Abstract

Loneliness and social isolation markedly increase mortality risk, and are linked to numerous mental and physical comorbidities, including sleep disruption. But does sleep loss causally trigger loneliness? Here, we demonstrate that a lack of sleep leads to a neural and behavioral phenotype of social withdrawal and loneliness; one that can be perceived by other members of society, and reciprocally, makes those societal members lonelier in return. We propose a model in which sleep loss instigates a propagating, self-reinforcing cycle of social separation and withdrawal.

## Abstract (web summary)

Loneliness markedly increases mortality and morbidity, yet the factors triggering loneliness remain largely unknown. This study shows that sleep loss leads to a neurobehavioral phenotype of human social separation and loneliness, one that is transmittable to non-sleep-deprived individuals.

## Introduction

Human beings did not evolve to be alone^1^. Sociality plays a fundamental part in the wellbeing of *Homo sapiens*^2–4^. Conversely, social isolation and loneliness are known risk factors for premature death^4,5^, more so than being obese^6^. Individuals who feel socially isolated and alone also have higher rates of cardiovascular disease, alcoholism and suicidality^1^, physical diseases related to stress and compromised immune function^7^, and in later life, greater risk of degenerative dementia^8^. Importantly, loneliness has a self-reinforcing characteristic. If an individual is perceived as lonely, others will frequently disengage from interacting with them^9^, resulting in a compounding cycle of social isolation. The broad concept of an asocial phenotype therefore involves multiple features, including social distancing and withdrawal from others^10^, subjective feelings of social isolation and loneliness, and others socially avoiding you^11–14^.

Though numerous factors are associated with social isolation and withdrawal from inter-personal interactions, recent evidence suggests that insufficient sleep may be one such candidate. In rodents, social isolation impairs subsequent sleep quality and efficiency^15^. In humans, intrapersonal distress and self-reported loneliness are linked to worse sleep quality^16^, specifically lower sleep efficiency^7,17,18^, while active socializing is associated with better sleep quality^19^.

Although social isolation can result in sleep impairment, it remains unknown, in human or non-human species, whether the opposite is true: does sleep loss lead individuals to feel lonely, become less social, and enforce greater social separation from others? Moreover, the underlying neural mechanisms associated with such an asocial phenotype remain unexplored. Finally, whether this effect is bi-directional, such that others in society reciprocally perceive sleep-deprived individuals as lonelier, even less desirable to interact with, is similarly unknown.

Here, we test the hypothesis that  a lack of sleep leads individuals to enforce greater social separation from others. Moreover, we examine whether the relationship between sleep loss and loneliness is observed following very modest reductions in sleep quality, from one night to the next, in a micro-longitudinal study. We further test the prediction that such a profile of social separation is instigated by hypersensitivity of brain networks that warn of human approach, yet a converse reduction in activity within prosocial networks associated with the comprehension of another’s intentions. Finally, we examine whether this effect is bi-directional, such that people blind to the experimental context nevertheless rate sleep-deprived individuals as being lonelier and less desirable to socially engage with.

## Results

**Fig. 1:**
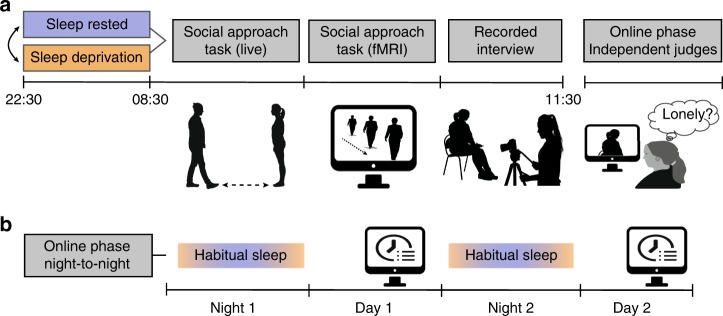
Experimental design. **a** In-laboratory repeated measures counterbalanced design. From left to right, the degree of social separation participants wished to keep from approaching others was assessed using a real, in-person human approach task, followed by a computerized version, used during a functional magnetic resonance imaging (fMRI) scan. At the end of each session, participants performed a recorded interview with open-ended questions, which was subsequently rated by independent judges, blind to study goal or sleep manipulation. **b** Online phase study design. Participants were asked to complete daily sleep logs for two consecutive nights tracking their habitual variations in sleep time. Following each sleep survey, participants completed a next-day questionnaire assessing social behavior and loneliness

The in-laboratory phase of the study involved 18 healthy adult participants enrolled in a counterbalanced, repeated measures study design involving two conditions: one night of sleep and one night of sleep deprivation (Fig. 1a and see Methods). In each condition, participants performed a standardized social distance task^20^, determining the degree of social separation they wished to keep from another person approaching them. Participants then underwent an fMRI scan to determine the neural correlates of social-approach sensitivity using a validated computerized version of the social approach task^21,22^. This task involved videos of real people and objects moving toward the camera, thereby rendering the neural correlates associated with socially relevant human-specific approach, and human-relative-to-object approach (see Methods). Following the scanning session, participants were filmed answering open-ended general questions as part of a structured interview.

Two online phases of the study were conducted through Amazon Mechanical Turk (MTurk). In the first, 138 participants were assessed across two nights and two subsequent days, sleeping as they chose (Fig. 1b). This first online study tested whether ecologically modest night-to-night variability in sleep quality (specifically, sleep efficiency^3,7,17,18,23–25^) predicted day-to-day changes in feelings of loneliness and social separation. We focused a priori on sleep quality, rather than sleep quantity, since loneliness^3,7,17,18,23–25^ and lower social engagement^19,26^ have repeatedly been associated with reduced sleep quality, rather than quantity^7,17,25,27^.

In the second online study, 1033 independent judges, blind to the experimental goals, viewed the structured interviews of the in-laboratory participants. The independent judges evaluated participants when sleep-rested and sleep-deprived on a range of socially relevant features (Fig. 1a, and see Methods). Judges were further asked how they themselves felt after watching each interview. Returning to the in-lab study, fMRI analysis focused a priori on several brain networks known to influence social interactions and dictate the social distance one chooses to keep from others. This act is known to require understanding and inferring the motives of other individuals^20,28^, as well as a neural mapping of peripersonal space that tracks the proximity of the approaching individual(s)^21,29^ (for review, see ^30^).

The former process of inferring mental states, potentailly leading to prosocial actions, reliably engages the theory of mind (ToM) network^31–34^, which includes the temporal–parietal junction and precuneus. Of relevance to social separation, participants who report feeling lonely show decreased activity within the ToM network in response to social stimuli, the magnitude of which correlates with higher subjective ratings of loneliness^35^. In addition, disorders of prominent social dysfunction, such as autism and schizophrenia, demonstrate impaired activity within this social cognition network^36,37^.

In contrast to prosocial attraction, the process of representing conspecific, potentially threatening, approach is mapped by the Near Space network^21,29^, which provides a warning signal of an advancing individual. The Near Space network has been identified using primate single unit recordings and involves regions of dorsal intraparietal sulcus and ventral premotor cortex that accurately track the approach of animate objects^21,29,38^. Related regions of the human dorsal intraparietal sulcus and ventral premotor cortex demonstrate equivalent increases in fMRI activity in response to advancing humans^21,29^, and similarly predicts greater social separation from others^22^. fMRI analyses therefore focused a priori on activity changes within these two recognized networks to assess the neural correlates of social approach.

**Fig. 2:**
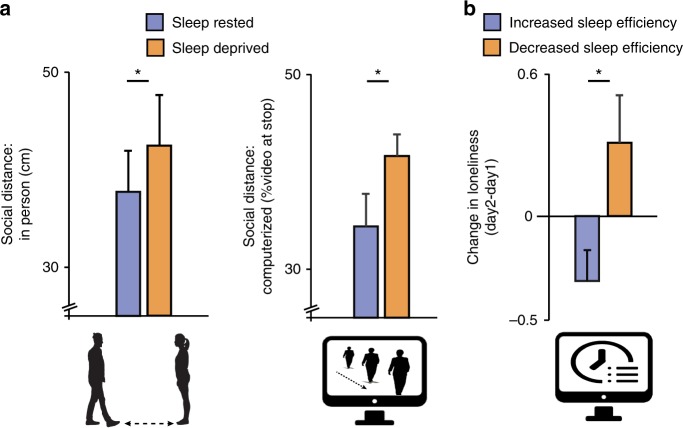
Behavioral results. **a** Significant increases in social distancing following sleep deprivation, relative to the sleep rested condition, for both the in-person (left) and computerized (right) social distance tasks (13.2% and 17.7% differences, respectively). **b** Night-to-night increases or decreases in sleep efficiency were associated with significant day-to-day increases and decreases in loneliness, respectively. *P ≤ 0.05; error bars reflect standard error of the mean

Focusing first on behavioral measures, and supportive of the social withdrawal hypothesis, participants in the in-laboratory study enforced greater social separation from others following one night of sleep deprivation for both the in-person approach task and the computerized task version (mean change 4.7 ± 2.1 and 5.76 ± 2.8 respectively, all *P* ≤ 0.05, Fig. 2a). For relative reference, the magnitude of these increases in inter-personal distance constitute approximately one-third of those reported in markedly asocial disorders, such as autism and schizophrenia^22,39^. In accordance with previous studies of loneliness^1,8,27,40–42^, the relative increases in social distance following sleep deprivation were not significantly associated with alterations in anxiety or mood (all *P* > 0.3, see Supplementary Note 1)—that is, the impact of sleep loss on increased loneliness was independent of any influence on mood or anxiety.

The MTurk experimental assessment offered a complementary test of the hypothesized relationship between sleep loss and loneliness, here focusing on ecologically modest night-to-night variability in sleep efficiency in a micro-longitudinal study within-subjects^3,7,17,18,23–25^. Results demonstrated that those individuals that suffered a reduction in sleep efficiency from one night to the next reported a corresponding next-day increase in loneliness from one day to the next, and vice versa (mean change 0.3 ± 0.19 and −0.26 ± 0.13, respectively, *P* < 0.05, Fig. 2b). Consistent with previous results^3,7,17,18,23–25^, and the in-laboratory findings, sleep efficiency remained a significant predictor of higher loneliness when controlling for the effects of mood and anxiety across days (*P* < 0.05, see Supplementary Note 1). Furthermore, the proportional change in night-to-night sleep efficiency predicted the corresponding day-to-day change in loneliness (R = −0.20, *P* < 0.05 see Methods). Therefore, both the acute (in-laboratory) manipulation of total sleep deprivation, and the more subtle changes in night-to-night sleep quality in the longitudinal study, causally triggered corresponding  changes in loneliness and social withdrawal.

**Fig. 3:**
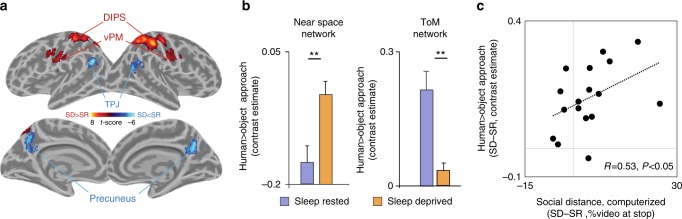
fMRI results of the in-lab study. **a** and **b** Significant clusters of activation within a priori regions of interest (ROIs) during human-relative-to-object approach, showing sleep-deprivation-related increases in activity in regions of the Near Space network (red), yet sleep-loss-related decreases in activity in regions of the Theory-of-Mind network (blue; all *P* < 0.05, FDR corrected). **c** Increased reactivity (human > object approach) within the Near Space network was positively associated with greater social distance separation following sleep deprivation. This relationship was not significant for activity in regions of the Theory-of-Mind network. **P < 0.005; error bars reflect standard error of the mean; gray lines reflect zero crossings. DIPS—dorsal intra-parietal sulcus, vPM—ventral premotor region, TPJ—temporal–parietal junction, SD—sleep deprivation, SR—sleep rested

Returning to the in-laboratory study, analysis of fMRI data demonstrated a bi-directional impact of sleep loss on brain activity during social approach in our a priori regions of interest (Fig. 3a–b). Sleep deprivation led to a significant decrease in activity within the theory-of-mind network associated with understanding the intentions and actions of another, yet a converse increase in reactivity within the Near Space network that warns of an advancing human^21,29^ (mean change −0.15 ± 0.04 and 0.13 ± 0.02, respectively, all *P* < 0.005). This sleep-deprivation increase in reactivity within the Near Space network was similarly significant when examining the contrast of human-only approach^43,44^, activity that focuses exclusively on a conspecific *(P* < 0.005, see Supplementary Note 2). Evincing a brain–behavior association, this sleep-deprivation increase in reactivity within the Near Space network further predicted the corresponding relative increase in social distance separation caused by sleep deprivation (Fig. 3c, *R* = 0.53, *P* < 0.05; this relationship was not significant for the ToM network, *P* > 0.5).

Together, these results indicate that a lack of sleep leads individuals to become more socially avoidant, keeping greater social distance from others. They do not, however, address whether other people conversely view sleep-deprived individuals as being lonelier and less desirable to engage with. This was addressed by the final online (MTurk) experimental phase of the study.

**Fig. 4:**
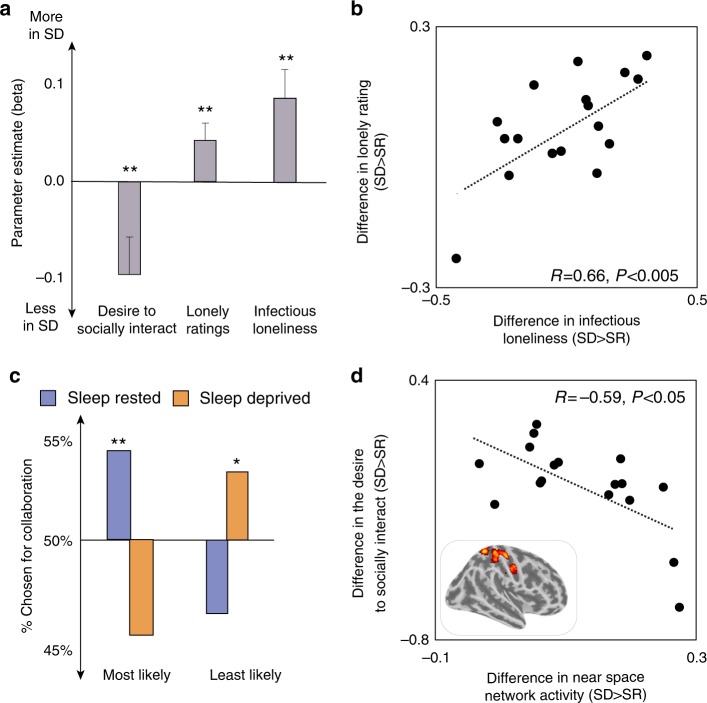
Ratings of independent judges, blind to the experimental conditions. **a** Change in social ratings of sleep-deprived participants (relative to their ratings in the sleep-rested condition) by independent judges (see Table S1). Following sleep deprivation, participants were rated as being significantly lonelier and less desirable to interact with. Judges also indicated feeling significantly lonelier themselves after watching videos of sleep-deprived participants. **b** Lonely ratings were positively associated with judges’ corresponding feeling of loneliness: the lonelier a judge rated an individual in a video, the lonelier they themselves sequentially felt. **c** Judges were significantly more likely to choose a sleep-rested participant to collaborate with compared to chance and significantly more likely to choose a sleep-deprived participant as their least favorite option for collaboration. **d** The sleep-deprivation increase in reactivity within the Near Space Network (human-only approach) of the experimental participants, correlated with the decreasing desire of judges to socially interact with these sleep-deprived individuals. This relationship was not significant for judges’ rating of perceived loneliness. *P < 0.05, **P < 0.005; correlations remain significant without each bottom outlier data point (Fig. 4b: *R* = 0.5, *P* < 0.05, Fig. 4d: *R* = −0.52, *P* < 0.05); error bars reflect standard error of the mean. SD sleep deprivation, SR sleep rested

Here, independent judges blind to the experimental conditions (*n* = 1033) rated sleep-deprived participants as being significantly lonelier, relative to the sleep-rested state, with ratings differences also observed across additional socially relevant factors (Fig. 4a, all *P* < 0.05, mixed model analysis, see Table S1). Moreover, judges rated themselves as feeling significantly lonelier after having viewed a video of a participant when sleep-deprived compared to sleep-rested, despite being blind to the experimental purpose. Indeed, the lonelier a judge rated an individual in a video, the lonelier they themselves subsequently felt (Fig. 4b, *R* = 0.66, *P* < 0.005). This indicates  that perceiving others as lonely can in itself trigger the transmission of loneliness from a sleep-deprived individual to a non-sleep-deprived other. As was the case with results from the in-laboratory experiment, judges ratings in the online MTurk study were not associated with participants’ change in mood or anxiety following sleep deprivation (*P* > 0.2, see Supplementary Note 1).

Consistent with a detrimental impact of loneliness on prosocial interactions, judges also indicated that they were significantly less likely to interact and collaborate with an individual in the video when that person was sleep deprived, relative to when they were sleep rested—this despite having no knowledge of this experimental manipulation (Figs. 4a, c, mean change in the desire to interact −0.08 ± 0.03 and 8% drop in collaboration choice, all *P* < 0.05). Demonstrating a neural association, the sleep-deprivation increase in Near Space network reactivity of in-lab participants, correlated with the decreasing desire of judges to socially interact with those individuals (Fig. 4d, *R* = −0.59, *P* < 0.05; human-only approach). However, this same measure of brain activity did not predict participants’ loneliness as rated by the judges (*P* > 0.5).

## Discussion

Taken together, these data establish that a lack of sleep—both total sleep deprivation and more modest, real-world reductions in sleep quality—leads to a behavioral profile of social withdrawal and loneliness. The underlying neural mechanism of this sleep-deprivation effect involves hypersensitivity in brain regions that warn of human approach—a social repulsion signal, yet impairment in regions that encourage  understanding of another’s intent, a prosocial signal. Of ecological relevance, individuals who know nothing of the experimental context nevertheless judge sleep-deprived participants as being significantly lonelier, and choose not to socially or collaboratively engage with them. Additionally, these judges consequentially feel significantly lonelier themselves after having viewed a video of a participant when sleep-deprived compared to sleep-rested. Indeed, the lonelier a judge rated an individual in a video, the lonelier they themselves subsequently felt.

An interesting question concerns the inner social motivation of our in-lab participants. The behavioral results established a clear decision by sleep-deprived individuals to impose greater social distance from others; the magnitude of which was predicted by corresponding changes in neural network activity. These findings indicate a conscious choice to enforce, and outwardly express, greater social separation from others. However, it is possible that individuals when sleep deprived still internally wish for as, if not more, social interaction than when they are sleep rested. Simply that there are other factors that make them outwardly act against this still-present social desire when sleep deprived. The in-lab study did not probe individuals as to whether they still maintained an inner desire for social connection, contrary to their overt decision not to do so. Our study therefore does not disambiguate this possibility of greater desire for social engagement, despite the outward choice against it (and the consequential social isolation and loneliness consequences of that overt choice). Nevertheless, the end-outcome remains the same—sleep-deprived individuals choose to socially withdraw, and furthermore, other members of society perceive those individuals as lonelier, and less desirable to engage with.

Numerous factors are known to affect social behavior and loneliness, including socioeconomic status, employment status, age, and sex^45^. Because of the within-subject design across all three studies (in-lab, Online study 1, and Online study 2) as well as their short time duration, the influence of these factors was limited or obviated. As a result, the changes in social distancing and loneliness observed following sleep loss are unlikely to be explained by variations in these demographic factors.

Low mood and increased anxiety can be comorbid with social separation and loneliness^18,40^. Both mood and anxiety changed as a result of sleep loss in our studies, and could have been instigating factors influencing social distancing and loneliness, rather than sleep loss itself. Counter to this possibility, however, control analyses demonstrated that the effects of sleep loss on neural and behavioral changes in loneliness and social distancing remained significant when taking into account co-occurring changes in mood and anxiety (see Supplementary Note 1). Such findings do not dismiss the influence of these affective factors on loneliness. Rather, our findings demonstrate that sleep loss significantly contributes to a profile of social withdrawal and loneliness independent of these co-occurring affective changes. Moreover, our findings are consistent with extant literature establishing loneliness as a distinct state from mood and anxiety^8,27,40–42^.

More generally, our findings establish that the state of sleep loss should be recognized as a social repellant, enforcing greater inter-personal separation on both sides of the social interaction. Additionally, we show that the asocial impact of sleep deprivation can propagate: people who come in contact with a sleep-deprived individual, even through a brief one-minute interaction, feel lonelier themselves as a result, indicating viral contagion of social isolation caused by sleep loss.

Our new series of findings motivate important next-step questions, particularly how these associations may change as a function of sex or as a function of age across the lifespan. For example, social withdrawal early in life serves as a significant predictor of loneliness in later adolescent and adult phases of life^11–14^. Moreover, older age is associated with marked changes in sleep quality^46^ and a significant increase in loneliness^47^. Future studies that explore the relationship between sleep changes across the lifespan and social withdrawal and loneliness are now especially relevant. Finally, our study raises the question of whether  the marked decline in sleep time throughout developed nations^48^ and the rising rates of loneliness in these same societies^1^ is simply co-ocuring, or instead, causally inter-related.

## Methods

### Participants of the in-lab experiment

Eighteen healthy adults, aged 18–24 years (mean: 20.2, s.d. ±1.5 years, nine women), completed a repeated-measures cross-over design (described below). Participants abstained from caffeine and alcohol for 72 h before each study session. Participants’ habitual sleep–wake rhythm was monitored for the three nights prior to study participation verified by sleep logs and actigraphy (a wristwatch movement sensor, sensitive to wake and sleep states). Exclusion criteria, assessed using a prescreening questionnaire, included: a history of sleep disorders, neurologic disorders, closed head injury, Axis 1 psychiatric disorders (according to the DSM-V criteria), history of drug abuse, and current use of anti-depressant or hypnotic medication. Subjects who reported sleeping less than 7 h per night or consuming three or more daily caffeine-containing drinks were also excluded from entering the study. No participant was excluded from the study due to poor sleeping patterns. Mean sleep duration of participants ranged from 7 h to 9.8 h of sleep according to actigraphy data (mean 8.2 h ± 0.71), with similar means obtained from daily sleep logs (8.38 ± 1.4).

The study was approved by the local human studies committee of the University of California Berkeley, with all participants providing written informed consent. Participants provided additional consent for the use of their filmed interview (detailed below) in future studies.

### In-lab experimental design

Following successful completion of screening, participants entered a repeated-measures study design (Fig. 1a), containing two sessions performed in a counterbalanced order: one after a normal night of sleep, and one after 24 h of total sleep deprivation. This within-subject design additionally served to minimize participant-related factors that can themselves impact social behavior and loneliness (e.g., age, sex, socioeconomic status, employment status)^1^. This design allowed for the assessment of changes in social distancing and loneliness resulting from the manipulation of sleep, while holding these demographic  factors of social influence fixed within individuals, over a short experimental time window.

In the sleep-deprived session, participants arrived at the laboratory at 9:30PM and were continuously monitored throughout the enforced waking period by trained personnel. During the sleep deprivation period, subjects engaged in a limited set of activities such as internet, email, short walks, reading, watching movies, or playing board games. The next morning at 9:00AM, participants completed mood and anxiety questionnaires (details below) followed by an in-person (live) social distance task assessment. At 10:30AM (±45 min), participants completed a computerized version of the social distance task inside the MRI scanner, followed by additional computerized social distance measures, outside the scanner.

In the sleep-rested session, participants arrived at the lab at 7:00PM and were wired up for an ambulatory polysomnography recording (detailed below), after which they were sent home, allowing for more naturalistic sleep. The next morning, participants returned to the laboratory, and had the electrodes removed. Participants then performed the same activities as those described above in the sleep deprivation condition, starting at the same circadian time. The sleep-rested and sleep-deprived sessions were separated by at least 7 days, with their order counterbalanced across participants.

### Social distance task—in-person

As in previous studies, social distance was measured using both a validated in-person interaction, and a computerized task version^20,21^. For the real, in-person task, an experimenter and the participant began by facing each other at a 3-m distance. Participants were told that the experimenter will slowly walk toward them. The participant then states “stop” when the experimenter gets to a distance that they would normally keep from a stranger. This measured separation is denoted as the comfortable distance and recorded using a digital laser measurer (Bosch GLM 50C). Thereafter, the experimenter continues to move toward the participant, and the participant is asked to say “stop” again at a distance that makes them feel uncomfortable. This separation reflects the uncomfortable distance, which is taken as the limit of one’s tolerance for human social proximity. The procedure was then repeated, but now the participant walked toward the experimenter, again stopping at both comfortable and uncomfortable distances. This provided social distance measures for being approached and for approaching others^20,21^. Following these bidirectional assessments, the task was repeated, but with an experimenter of the opposite gender. The average of both approaching and being approached, by a male and a female, is used as the outcome measure^20,21^. Different experimenters were used in each study condition (sleep rested or deprived) to avoid any familiarity effects, with experimenters randomized in assignment across participants and conditions.

### Social distance task—computerized (fMRI)

A computerized version of the social distance task, with known neural correlates and sensitivity^21,22^, was used during the fMRI scan. Here, participants viewed experimentally controlled videos of 24 individuals walking toward a camera at a steady pace and with a neutral expression (*human approach*). These 24 individuals were a mixture of races and ethnicity, mean age 21.6 years, 12 males and 12 females. Individuals in the videos started 3 m away from the camera lens, and then walked at a standardized cadence toward the camera, eyes directly fixed on the camera lens, and stopped approximately 3 cm from the lens. Each video lasted 15.3 s on average (±1.8 s). These video stimuli allowed for the creation of an fMRI contrast focused exclusively on an approaching conspecific (human-only approach)^28,43,44,49^. Additional videos of equivalent duration were also created for inanimate, non-threatening objects. Examples included a lamp, a basket and a tray. In these videos, objects started out in center-screen, small size, and gradually increased in size as if getting nearer (object approach). These video stimuli provided an on-task comparison with the human approach videos, allowing for an alternate brain contrast comparing two forms of item engagement (human approach > object approach)^21,22^.

During the fMRI scanning session, all videos played from start to finish, rather than having the participants stop the videos. This prevented any possible differences in stimulus exposure duration for each fMRI trial, across conditions.

Half of the object and human video trials were presented in the sleep-rested condition, the other half presented in the sleep-deprivation sessions, with the version order counterbalanced across participants. The videos were presented in two runs, with each run containing a total of 12 videos. The different video trials (humans, objects) appeared in randomized order within each run.

In order to verify attention to each video, participants answered a multiple-choice information question (e.g., what was the color of the tray? What was the person’s hair color?). The question was presented for 8 s following each video, followed by an inter-trial fixation period (jittered, 4–8 s). The start of each run contained a 10-s fixation block, to allow for steady-state equilibrium of the BOLD fMRI sequence.

Noted above, all video trials during fMRI scanning played from start to finish to standardize stimulus exposure across trials and conditions. Immediately after the scan, however, participants viewed all videos again but were required to press the spacebar to stop the video when the approaching stimuli made them “feel uncomfortable”^20,49^. During this part of the task, participants sat in front of a computer screen at a fixed distance of 30 cm. The stop duration determined by the spacebar press, relative to the length of the video, was used as a computerized measure of social distance (i.e., 100% would indicate maximal distance from the approaching figure). This provided a homolog measure to the real, in-person version of the task (above). Confirming that the computerized measure was sensitive to in person social distance aversion, these two measures were significantly correlated with each other across individuals and conditions (*R* = 0.58, *P* < 0.05).

### Filmed Interviews

After the scanning session in both the sleep-rested and sleep-deprived conditions, participants performed a “speak freely” interview that was filmed^50^. These interviews were subsequently used by independent judges, asked to evaluate the participants on socially relevant characteristics (see below). During the interview, the experimental participants sat in front of a microphone facing the interviewer and a video camera. Participants were then asked to give their opinion on 20 different themed questions (Table S2, e.g., Do you think everyone should go to university?), with ten different questions being asked at each of the sleep sessions. The ten questions were counterbalanced in order and use across conditions and participants. The response from participants were required to be approximately 1 min in duration to provide a robust duration of filming for subsequent use. If participants stopped short of 1 min, the interviewer prompted them to say more by using open-ended follow-up questions (e.g., “why do you think that?”). For use in the subsequent online ratings by independent judges, the interviews were edited to create shorter clips  (mean duration 67.2 ± 17.4 s), each including one question.

### Loneliness and habitual variations in sleep (Online study 1)

In addition to the in-lab total sleep deprivation study, Online study 1 tested whether more modest night-to-night variability in sleep quality, focusing a priori on sleep efficiency, predicted day-to-day changes in feelings of loneliness and social separation. Similar to the in-lab design, we focused on changes within an individual from one day to the next. This within-subjects, repeated-measure design minimized the  influence of non-sleep related factors on the outcome measures of social withdrawal and loneliness (e.g., sex, age, employment status, socioeconomic status). This was similarly true for controlling inter-individual idiosyncratic biases in subjective sleep estimates and measures of social withdrawal and loneliness. Since the goal of the study was to test whether ecologically naturalistic changes in sleep quality are sufficient to inflect next-day changes in loneliness, we deliberately did not provide instructions to subjects to curtail or elongate their sleep. Online study 1 therefore served as an important complement to the in-lab total sleep-deprivation manipulation, while keeping a common within-subjects, repeated measures assessment design.

We focused a priori on changes in sleep quality, since lower sleep quality, whether measured objectively or subjectively, has been shown to be a robust marker of higher loneliness^3,7,17,18,23–25^, and lower social engagement^19,26^. This focus is further supported by the fact that daily variations in sleep quantity (duration) show weaker associations with loneliness^7,17,25,27^, than sleep quality.

A total of 293 participants (mean age 36.61 years, 50% women) were recruited for this study phase using MTurk—a platform where individuals can perform online tasks for a specified reimbursement (here, $1.80). Enrollment was restricted to those with IP addresses in the United States, and a prior online MTurk approval rating of 95% or higher. Following recruitment, participants were asked to complete sleep surveys quantifying their sleep across two consecutive nights (see Table S3), followed by next-day assessment of how lonely they felt using a short form of the validated Revised UCLA Loneliness questionnaire^51^. Participants were also asked to report their daily mood and anxiety at each daily survey (detailed below) as well as report how much of their day they had spent with others (ranging from 0 to 100%; see Supplementary Note 3) to further examine changes in social withdrawal. All questions were presented in random order.

Providing a reasonable daytime period for social interactions, and to ensure that the assessment of loneliness was performed within a restricted time, the survey was available online during a specific opportunity window in the evening, starting at 6PM PST until day’s end. Due to the more longitudinal, multi-day nature of this online study, there was an expected higher dropout rate: 138 of the 293 recruited participants ended up completing both sets of measures across the 2 days. Additionally, four participants were excluded from the daily surveys due to duplicate entries of their survey data (e.g., completed the same daily survey multiple times).

As noted above, analysis focused a priori on sleep quality, given previous studies linking loneliness with measures of sleep efficiency^7,16,17^ rather than duration^18^. Moreover, subjective estimates of sleep efficiency are objectively accurate, and do not differ significantly from objectively determined polysomnography sleep measures^52,53^.

Sleep efficiency was calculated using participants’ daily sleep surveys, based on the percent of time asleep out of total sleep duration (i.e., total sleep time minus sleep latency and time spent awake after sleep onset^54^). Using this measure, we tested whether night-to-night variability in sleep quality within participants predicted day-to day changes in feelings of loneliness. Only participants with valid sleep log entries on both days were included in the analysis (*N* = 138, mean age = 37.6, 52% women). As noted above, the in-lab study involved a binary sleep condition (sleep rested, sleep deprived). To provide a complementary within-subjects design in Online study 1, participants were dichotomized on the basis of an increase or decrease in sleep efficiency from one night to the next (above or below 0% change, *N* = 75 and *N* = 56, respectively). Thereafter, a comparison of the corresponding change in loneliness within individuals, from one day to the next, was performed (Fig. 2b).

In addition to this dichotomized approach, we further tested our hypothesis in Online study 1 using regression analysis, treating the data as a continuous variable set. Similar to the categorical analysis results, the change in night-to-night sleep efficiency significantly correlated with the change in corresponding day-to-day loneliness (*R* = −0.20, *P* < 0.05), such that a reduction in sleep efficiency resulted in a corresponding increase in next-day loneliness.

### Independent judges and online ratings (Online study 2)

To examine the bi-directional nature of our hypothesis, we additionally tested the prediction that other individuals, unaware of the study context, would rate participants as being lonelier and less desirable to engage with when sleep deprived, relative to when sleep rested. A total of 1033 online judges (mean age 35.4 years, 52% women) were recruited using MTurk with similar enrollment criteria as described above. Individuals blind to the experimental purpose and participant condition viewed the interview videos of the in-laboratory participants, and rated these participants across a number of social categories (see Table S1 for details). In addition, judges themselves were asked whether they felt lonelier as a consequence of having engaged with the participant in each video.

Each judge was asked to watch four short videos in the same survey and provide ratings on each one. Online videos were of a duration known to be sufficient to produce measurable changes in inter-personal social dynamics^55,56^ (~1 min). Moreover, this shorter video duration also allowed for a controlled length of social interaction that did not involve excessive time-on-task that may introduce confounds, such as declining attention and non-compliance of viewing.

The videos were pseudo-randomized across judges, so that each judge always viewed an equal number of experimental conditions (i.e., two sleep-rested and two sleep-deprived videos) and never the same participant more than once. Having raters evaluate both sleep-rested and sleep-deprived participants in the same short session controlled for any intra-individual differences in judges’ mood state, or inter-individual idiosyncratic biases. In this way, rater bias would be applied evenly to the sleep-rested and sleep-deprived individuals they viewed, and could not explain differences in ratings between the sleep-rested and sleep-deprivation conditions. Furthermore, our mixed model analysis (detailed below) also included the factor of “judges” as a random variable, thereby further controlling for any inter-individual rater bias. In addition to these four videos (two sleep-rested and two sleep-deprived), a fifth video, featuring a non-experimental participant, was inserted for practice purposes at the beginning of each survey to allow judges to become familiar with the rating process, and was not included in the analysis.

Following each video, judges were asked to answer 13 socioemotional- and fatigue-related questions regarding the person they viewed (see Table S1, e.g., How lonely do you think this person is?). All questions were presented in random order, immediately following each video. Of relevance to fatigue, we deliberately chose not to ask judges to rate how sleepy participants appeared so as to avoid explicitly revealing the premise of the study, keeping judges blind to the experimental sleep manipulation. Instead, judges were asked to rate the experimental participants on a scale of low–high energy. Our pilot data demonstrated that this construct of energy, reflecting how active/alert a subject was, negatively correlated with sleepiness as measured by the Karolinska sleepiness scale (*N* = 42, *R* = −0.64, *P* < 0.0001; see Supplementary Note 4). That is, the rating of energy provided a reliable surrogate of perceived sleepiness.

All socioemotional- and fatigue-related ratings were based on a four-point scale (e.g., “not at all lonely”; “somewhat lonely”; “quite lonely”; “very lonely”). The exception to this rating scale was the social interaction question, which included a fifth, “no preference”, option. Finally, after viewing these videos, judges were presented with pictures of all four participants they had watched. The judges were asked to select which individual they would choose to collaborate with if they were performing a co-working project, from most likely to least likely. One of the 1033 judges was excluded due to identical button-response selection for all questions. For the collaboration question analysis, 34 out of the 1033 judges were excluded due to online technical problems with presentation of the picture-response questions. Our a priori analyses of judges’ ratings focused on three key measures: (1) loneliness, (2) infectious loneliness, and (3) desire to socially interact, in accordance with the overarching hypothesis of the study regarding sleep induced changes in loneliness and social withdrawal. In addition to the mixed model analysis of judges’ ratings (detailed below), loneliness scores across judges were averaged for each experimental participant. A similar mean-score approach was used for the measure of infectious loneliness (i.e., how lonely that participant made the judges feel). These participant-mean values were then averaged for the sleep-rested and sleep-deprived conditions separately, and statistical comparisons made between the two conditions.

### Statistical analyses

Analysis of the behavioral ratings across the sleep-rested and sleep-deprivation conditions used planned comparison, two-tailed paired *t* tests (*α* 0.05). For the MTurk ratings by the independent judges across the range of socioemotional questions, a mixed effects linear regression model was used, with two random effects in order to take into account variations in both (1) participants and (2) judges. The fixed effect was the experimental condition, wherein sleep rested represented the intercept and the slope being the effect of sleep deprivation. Analysis of the collaboration question was performed using a binomial distribution test, with a 50% chance of choosing a sleep-rested or -deprived participant to collaborate with.

### fMRI acquisition

Blood oxygenation level-dependent contrast functional images were acquired with echo-planar T2*-weighted (EPI) imaging using a Siemens 3 Tesla MRI scanner with a 12-channel head coil. Each image volume consisted of 37 descending 3.5 mm slices (96 × 96 matrix; time to repeat(TR) = 2000 ms; time to echo (TE) = 22 ms; voxel size 3.5 × 3.5 × 3.2 mm, flip angle = 50°, 0.3 mm interslice gap). One high-resolution, T1-weighted structural scan was acquired at the end of each session (256 × 256 matrix; TR = 1900; TE = 2.52; flip angle = 9°; FOV 256 mm; 1 × 1 × 1 mm voxels).

### fMRI analysis

Preprocessing and data analysis were performed using Statistical Parametric Mapping software implemented in Matlab (SPM12; Wellcome Department of Cognitive Neurology, London, UK). Images were motion corrected and slice time corrected, and then spatially normalized to the Montreal Neurological Institute template and smoothed using a 6-mm full-width-at-half-maximum (FWHM) Gaussian kernel using default parameters in SPM12. For each subject, trial-related activity was assessed by convolving a vector of trial onsets with a canonical hemodynamic response function.

The six movement-related covariates (three rigid-body translations and three rotations determined from the realignment preprocessing step) were used as regressors in the design matrix for modeling movement related artifact in the time series. To further address the influence of motion on BOLD data, we calculated frame-wise displacement (FD) of head motion based on the motion parameters estimated during preprocessing using the ArtRepair toolbox^57^. TRs including FD values larger than 0.5 were interpolated with the nearest artifact-free TRs surrounding the motion. Subjects were excluded from analysis if both movement regressors and FD values were larger than 2 mm. Due to such movement of artifacts, one subject was removed from further analyses.

To control for physiological noise, five principal components of cerebrospinal fluid (CSF) and white matter signal were added as regressors to the design matrix, implemented through the CompCor pipeline^58^. Extraction of white matter/CSF signal was derived using probabilistic maps segmented from the T1-weighted anatomical image of each participant using the segment function implanted in SPM12. Masks were then thresholded at a probability value of 0.99 for white matter and 0.95 for CSF, converted to functional resolution and eroded to eliminate isolated voxels.

Following pre-processing, a general linear model (GLM)^59^ was specified for each participant to investigate the effects of interest. Contrasts were created at the first level focusing on human vs. object approach contrast to target regions sensitive to social approach. The resulting contrasts were then taken through to a second level, random effects analysis to assess group-level effects, examined using a paired *t*-test (Sleep Rested < > Sleep Deprived). Analyses focused a priori on activity within two functional networks associated with social approach: the Near Space network, encapsulating the ventral premotor (vPM) regions and the dorsal intra-parietal sulcus (DIPS), and the ToM network, encapsulating the precuneus, temporal parietal junction, and medial frontal cortex^21,60^. Regions of interest (ROIs) were an independent set of literature-defined regions, constructed as 12 mm spheres centered around reported coordinates of each network separately (see Tables S4 and S5 for non a priori whole-brain exploratory analysis).

Condition differences in ROI activity were reported using a threshold of *P* < 0.05 corrected using False Discovery Rate (FDR; spatial threshold of ≥5 voxel cluster), to account for multiple comparisons^61,62^. Average activity for each network was extracted from the full 12 mm ROI spheres. Comparisons of average ROI activity between the two conditions were analyzed using a two-tailed paired *t* test (*α* 0.05). Associations between the average ROI activity and behavioral measures of social distance were tested using Spearman’s correlation.

### Mood and anxiety assessment

Mood and anxiety have both been linked to loneliness^40,63^ as well as to lack of sleep^64^. We therefore controlled for these affective changes in both the online and in-lab studies. In the in-lab study, changes in mood and anxiety states were assessed at 9AM on the experimental mornings using the Positive and Negative Affective Scale (PANAS^65^) and the State-Trait Anxiety Inventory (STAI^66^), respectively. In Online study 1, changes in mood and anxiety were tracked in each daily measure using the short form of the PANAS^67^ and STAI^68^ questionnaires.

### Sleep recordings

Sleep was recorded by standard polysomnography including electroencephalography, electromyography, and electrooculography recordings. Electroencephalography was recorded from nine scalp electrodes (F3, F4, F7, F8, C3, C4, P3, P4, and Oz; International 10–20 System), with a central reference. EEG signals were filtered at 0.15–35 Hz and sampled at 200 Hz. Polysomnographic recordings were scored according to standard criteria^69^. Sleep statistics are provided in Table S6, and conform to population norms for this age range^70^.

### Data availability

The data that support the findings of this study are available from the corresponding authors upon request.

## Electronic supplementary material

Supplementary Information
