# Supplementary material for: Sleep loss causes social withdrawal and loneliness
Source: Nat Commun. 2018 Aug 14;9:3146. doi: 10.1038/s41467-018-05377-0 (PMC6092357; doi:10.1038/s41467-018-05377-0)
Supplement: Supplementary file 1 — Supplementary Information [file 41467_2018_5377_MOESM1_ESM.pdf]

Supplementary Information: Sleep Loss Causes Social Withdrawal and Loneliness

Eti Ben Simon and Matthew Walker

Supplementary Tables:

Supplementary Table 1: Judges Ratings of in-lab participants in Online study 2

Supplementary Table 2: Filmed Interview Questions

Supplementary Table 3: Daily Sleep Log

Supplementary Table 4: Regions of Interest for fMRI analysis

Supplementary Table 5: Exploratory whole brain analysis

Supplementary Table 6: Polysomnography sleep characteristics for the sleep-rested night

Supplementary Notes:

1. Anxiety and Mood assessments in relation to Social Withdrawal and Loneliness
2. Near Space network activity for human-only approach and its relation to behavior
3. Sleep Efficiency and changes in Social Withdrawal and Loneliness
4. Perceived Energy and changes in social perception by external judges

Supplementary Figures:

Supplementary Figure 1: Mood and Near Space network activity

Supplementary Tables

Supplementary Table 1: Judges Ratings of in-lab participants in Online study 2

Rated factor	Sleep rested (s.e.)	Effect of sleep deprivation (s.e.)	p-value
**Desire to socially interact***	3.37±0.07	-0.08±0.03	0.005
**Lonely***	1.53±0.05	0.08±0.02	0.001
**Infectious Loneliness***	1.25±0.02	0.05±0.01	0.001
Threatening	1.16±0.01	-0.004±0.01	0.9
Intelligent	2.81±0.04	-0.03±0.02	0.1
Anxious*	1.76±0.05	0.07±0.03	0.01
Funny*	1.83±0.03	-0.04±0.02	0.04
Healthy*	3.03±0.05	-0.06±0.02	0.02
Perceived Energy*	2.3±0.07	-0.15±0.02	0.0001
Trustworthy	2.99±0.03	-0.0002±0.02	0.9
Self-Centered	1.7±0.17	-0.002±0.02	0.9
Feeling Uncomfortable	1.35±0.02	0.03±0.02	0.07
Friendly	3.03±0.04	-0.04±0.02	0.06

*Betas and standard errors derived from a mixed effects linear regression model with sleep as the fixed effect and subject\rater as two random effects. Desire to socially interact, loneliness and infectious loneliness served as a priori analyses of interest. The remaining ten ratings were analyzed post-hoc to confirm selectivity of the observed effects.

Supplementary Table 2: Filmed Interview Questions

**Version 1**	
Q1.	What do you think is a good strategy to help reduce poverty?
Q2.	What do you think about social media?
Q3.	Do you think everyone should go to university?
Q4.	What’s your opinion on self-driving cars? Are you excited to try them?
Q5.	What do you think about artificial intelligence?
Q6.	What’s your favorite place to visit?... What do you like to do when you get there?
Q7.	What’s your favorite film?
Q8.	Who’s your favorite musician?
Q9.	Can you tell us a little bit about your hobbies?
Q10.	Could you tell us something interesting you learnt in the past week?
**Version 2**	
Q11.	What’s your opinion on the status of women in the work force?
Q12.	What do you think smartphones will be like 10 years from now?
Q13.	Are you concerned about Genetically Modified food?
Q14.	What do you think about space exploration? and what about the search for extra-terrestrial intelligence?
Q15.	What do you think is the biggest challenge to human health?
Q16.	What was the best trip you ever took?... When was that?
Q17.	What’s your favorite book?
Q18.	Do you prefer to live in a big city or a small town?
Q19.	What do you look for in a good friend?
Q20.	Could you tell us something interesting you learnt in the past week?

*Questions were presented in a random order with question versions counterbalanced across participants and sleep conditions.

Supplementary Table 3: Daily Sleep Log

Sleep diary questions completed by participants of Online study 1 across two consecutive nights of habitual sleep

Q1.	What time did you go to bed last night?
Q2.	How long did it take you to fall asleep last night?
Q3.	What time did you wake up this morning?
Q4.	How many times did you wake up during the night?
Q5.	If you woke up during the night, how long did it take you to get back to sleep?

Supplementary Table 4: Regions of Interest for fMRI analysis

Theory of Mind ROIs

X	Y	Z	Laterality	Label	BA
1	58	19	Mid	superior frontal gyrus	9
2	62	18	Mid		
−4	56	8	Left	medial frontal gyrus	9
2	46	-19	Right		
−52	−56	24	Left	superior temporal gyrus	22
55	-53	24	Right		39
−3	−56	37	Mid	precuneus	31
2	−58	26	Mid	posterior cingulate	31
59	−18	−17	Right	middle temporal gyrus	21
-59	-26	-9	Left		

Near Space network ROIs

X	Y	Z	Laterality	Label	BA
-19	-69	47	Left	Superior parietal gyrus (dorsal)	7
-16	-53	67			
-34	-40	63			
-11	-48	63			
-22	-43	64			
-23	-46	69			
13	-49	64	Right	Superior parietal gyrus (dorsal)	7
29	-47	58			
40	-36	62			
34	-29	47			
-46	-7	27	Left	Precentral gyrus (ventral)	6
-52	-6	34			
47	-7	30	Right	Precentral gyrus (ventral)	6

*MNI coordinates

Supplementary Table 5: Exploratory whole brain analysis

Exploratory whole-brain analysis showing all peak activations (MNI coordinates, $P<0.001$, min 5 voxels for paired comparison; sleep rested <> sleep deprived) of the human vs. object approach parametric contrast. These non a priori whole-brain data are provided simply for descriptive purposes, without inference. Cluster size is in voxels; voxel size is $3mm^3$.

X	Y	Z	T	Anatomical Label	Cluster Size
**Sleep Deprived>Sleep Rested**					
X	Y	Z	T	Anatomical Label	Cluster Size
-42	-37	53	8.03	L Postcentral	263
-18	-67	50	7.28	L Sup. Parietal	29
51	8	20	6.62	R Inf. Frontal (operculum)	22
33	-49	59	6.24	R Sup. Parietal	156
27	-19	20	5.64	R Insula	13
45	17	-1	5.50	R Insula	6
-15	2	41	5.20	Mid cingulate cortex	11
3	-67	-13	5.20	Cerebellum	37
-57	-13	23	5.14	L Post Central	8
60	-19	35	5.13	R Post Central	20
30	-13	47	4.75	R Pre Central	21
6	35	53	4.47	R Sup. Medial Frontal	6
**Sleep Rested>Sleep Deprived**					
X	Y	Z	T	Anatomical Label	Cluster Size
-48	-64	26	6.23	L Angular	41
-42	-31	-16	6.14	L Inf. Temporal	5
45	5	-25	5.13	R Mid. Temporal	5
-3	-58	20	4.95	Precuneus	18
42	-58	20	4.52	R Mid. Temporal	15
6	-58	35	4.40	Precuneus	9

Supplementary Table 6: Polysomnography sleep characteristics for the sleep-rested night (mean ± SD)

	Time (min)	Percentage of total sleep time
**Sleep latency**	15.53 ± 13.07	
**Total sleep time**	403 ± 67.63	
**WASO**	26.85 ± 24.6	
**Sleep Efficiency**	88.97± 6.03	
**NREM stage 1**	31.5 ± 11.86	8.03 ± 3.21
**NREM stage 2**	202.41 ± 46.11	50.14 ± 7.65
**NREM SWS**	90.53 ± 33.06	22.99 ± 7.56
**REM**	78.58 ± 29	19.26 ± 6.17

WASO, wake after sleep onset; NREM, nonrapid-eye-movement sleep; SWS, slow-wave sleep (SWS, NREM stage 3 and 4); REM, rapid-eye-movement sleep.

Supplementary Notes

Supplementary Note 1.

Anxiety and Mood assessments in relation to Social Withdrawal and Loneliness

Sleep deprivation triggered an increase in state anxiety, indexed using the state-trait anxiety inventory (STAI) questionnaire, measured at 9AM on the morning of each session, prior to task performance and MRI scanning (M=33.52±1.9 to M=41.7±2.24; $P<0.01$). Positive and negative mood was also assessed at the same time using the Positive and Negative Affect Schedule (PANAS). Following sleep deprivation, there was a significant decline in positive mood (M=27.41±1.94 to M=16.88±1.59; $P<0.001$) and an increase in negative mood (M=14±0.64 to M=15.7±0.8; $P<0.05$), relative to the sleep rested condition. However, these changes in anxiety and mood were not significantly associated with the reported changes in social distance, for either the real, in-person task or the computerized task (all $P>0.26$).

Beyond social distance results, we further examined 3 additional key measures across our studies to fully address the selectivity and independence of our sleep-loss effects of social distance and loneliness from the effects of mood and anxiety. Specifically, we examined **1)** Brain activity from the in-lab study and its relation to mood and anxiety (focusing on our a-priori networks of theory of mind [ToM] and Near Space [NS]), **2)** Mood and anxiety ratings of participants from Online study 1 and their relation to sleep efficiency and loneliness, and **3)** External judges ratings from Online study 2 and their association with in-lab participants' change in mood and anxiety. Below we address each of these analyses separately.

I. Mood, anxiety and brain activity:

Beyond the association with social distance separation, we further examined whether the ancillary sleep-loss changes in mood and anxiety demonstrated similar associations with changes in brain network activity. Specifically, we examined the counterhypothesis that the sleep-loss reduction in ToM activity, and increase in NS network activity, were alternatively accounted for by reductions in mood and/or increases in anxiety, respectively. No such associations were found, and if anything, the inverse associations between brain activity and mood and anxiety were observed, relative to the direction of association with the increase in personal distance separation.

First, negative mood was inversely correlated with the change in ToM activity ($R=-0.6$, $P<0.05$), such that the greater the sleep-loss increase in negative mood, the smaller the change in ToM network activity. Second, a similar inverse association was observed for

anxiety, such that the greater the sleep-loss increase in anxiety, the less the sleep-loss change in ToM network activity (i.e., the more sleep-rested-like the profile of ToM activity; R=0.43, P=0.08). Third, within the NS network, the sleep-loss reduction in positive mood actually predicted less (rather than more) activity following sleep deprivation (R=0.55, P<0.05; see **Supplementary Figure 1**). Finally, sleep-loss-related reductions in negative mood, and increase in anxiety, actually predicted less change in NS network activity after sleep deprivation (R=-0.647, P<0.01; R=-0.53, P<0.05, respectively)—the opposite direction to that observed for increased social withdrawal, and the overall main effect of sleep loss on brain activity.

Taken together, these results do not support the counterhypothesis that the sleep-loss-related associations between brain and social distancing are alternatively accounted for by changes in mood and/or anxiety following sleep deprivation.

II. Mood and anxiety in Online study 1 (changes in habitual sleep and loneliness)

In Online study 1, participants were asked about their mood and anxiety during the completion of each daily sleep survey. Consistent with previous reports[1,2], there were co-linear relationships between loneliness, mood and anxiety across the daily measures: changes in loneliness from one day to the next were associated with increased negative mood (R=0.34, *P*<0.0001), increased anxiety (R=0.26, *P*<0.005) and lower positive mood (R=-0.189, *P*<0.05).

To further examine the impact of mood and anxiety on the reported association between sleep efficiency and loneliness a multiple regression model was created that includes the a- priori sleep variable—sleep efficiency, together with mood and anxiety scores as predictors of the key outcome variable of loneliness. Fitting previous findings[2-8], results of the multiple regression model demonstrated that sleep efficiency remained a significant predictor of loneliness when controlling for the effects of mood and anxiety (β=-2.3±1.1, *P*<0.05). These findings suggest that despite co-occurring changes in mood and loneliness, reduced sleep efficiency remains a predictive factor of higher loneliness.

To further validate that changes in sleep efficiency are not associated with changes in mood, we examined the association between mood and anxiety ratings of our online participants and the change in their sleep efficiency measures. Sleep efficiency was not significantly associated with the change in either positive mood (R=0.02, *P*>0.8) or negative mood (R= -0.106, *P*>0.2), nor with changes in anxiety (R= -0.02, *P*>0.7). These results were similarly true when using a binary measure for the change in sleep efficiency as described in the main text

($P>0.6$ for negative and positive mood, as well as anxiety, participants divided by an increase or decrease in sleep efficiency). Together, these results indicate that the relationship between loneliness and sleep efficiency is not parsimoniously accounted for by changes in mood or anxiety.

III. Mood and anxiety in and Online study 2 (Independent judges ratings)

Focusing on our three key outcome measures—ratings of loneliness, willingness to interact, and infectious loneliness, we examined the association between external judges' ratings (averaged per participant across sleep deprived, relative to sleep rested) and the corresponding sleep-loss changes (sleep deprived, relative to sleep rested) in mood and anxiety reported by those participants. None of the judges ratings for all of these key measures were significantly associated with the sleep-loss change in mood or in anxiety (all $P>0.2$). These findings suggest that the results reported in Online study 2 are not associated with participants' altered mood or anxiety following sleep deprivation.

In summary, our collective study findings suggest that disturbed sleep significantly contributes to a profile of social withdrawal and loneliness beyond co-occurring changes in mood and anxiety. The results of these control analyses are fitting with the extant literature, wherein loneliness has been demonstrated as a state distinct from mood and anxiety by measures of both statistical[1,9] and functional independence[10-13].

Supplementary Note 2.
Near Space network activity for human-only approach and its relation to behavior

The computerized version of the social distance task successfully activated the Near Space network, known to be sensitive to approach of both humans[14-16] and objects[17,18] into one's personal space. We therefore examined two contrasts of interest in Near Space network activity: 1) human specific approach ("human only"), and 2) human- relative to object-approach ("human>objects"). The latter contrast (described in Fig. 3) allowed us to confirm that condition differences (sleep rested < > sleep deprivation) in Near Space network activity were human-approach specific. The former contrast (described in Fig. 4) then provided a more specific examination of activity that focuses solely on an approaching conspecific[16,19]. Supporting the sensitivity of Near Space network to social approach, both contrasts revealed a significant increase in activity following sleep deprivation (Mean change for human>objects 0.13±0.08, human only approach 0.13±0.1, both $P<0.005$).

Since Near Space network activity was associated with both 1) computerized social distance and 2) judges ratings of the desire to socially interact (see Fig.3C and 4D respectively), we further examined whether these measures are associated with each other as a cautionary test. Importantly, there was no significant correlation between computerized social distance and the desire to socially interact (R=-0.33, P>0.15), suggesting that both social measures are independently associated with Near Space network activity.

Supplementary Note 3.

Sleep Efficiency and changes in Social Withdrawal and Loneliness

As noted in the main text, changes in sleep efficiency triggered an increase in ratings of subjective loneliness from one day to the next (see Fig. 2c). Beyond a within-subject change in loneliness we further examined the link between sleep efficiency and loneliness *across* participants, focusing on each daily measure separately. Consistent with previous reports[3,7,8,12], sleep efficiency was negatively correlated with loneliness on each of the respective days measured (R=-0.23 for day 1, R=-0.24 for day 2, both P<0.005).

In addition to the association between poor sleep efficiency and greater loneliness, we further evaluated whether increases in loneliness were associated with real-life social behaviors that are indicative of social withdrawal. To that end, participants reported how much of their day they had spent with other people, relative to being alone (ranging from 0 to 100%). Confirming a detrimental impact of loneliness on social withdrawal, changes in loneliness from one day to the next were associated with less time that participants spent engaging with other people (R=-0.191, P<0.05). Similar to the above finding, this result was similarly true across participants when analyzing each measurement separately (R=-0.27 for day 1, R=-0.28 for day 2, both P<0.005).

Finally, we examined whether sleep efficiency measured on the sleep-rested night in the in-lab study, demonstrated a similar directional relationship between sleep and social withdrawal to that of Online study 1, despite being a weaker test of this hypothesis since the former is an inter-individual test (unlike the intra-individual precision in Online study 1). Consistent with a detrimental impact of worse sleep on social withdrawal, we find that lower sleep efficiency on the sleep rested night predicted greater in-person social distance separation across our participants (R=-0.46, P=0.06).

Supplementary Note 4.

Perceived Energy and changes in social perception by external judges

As mentioned in the main text, we deliberately chose to avoid asking judges to rate how sleepy participants appeared in order to refrain from explicitly revealing the premise of the study and keeping judges blind to the experimental sleep manipulation. Instead, we asked judges to rate experimental participants on a scale of low---high energy. First, and confirming our sleep manipulation, sleep deprived participants were rated as significantly lower in energy compared to their sleep rested state (β=-0.154, P<0.0001, mixed model analysis). We further examined whether changes in perceived energy were associated with raters' desire to socially interact with in-lab participants or with judges loneliness ratings. First, the difference in judges ratings of participant loneliness (sleep deprived vs. sleep rested) were negatively correlated with the same participants' change in perceived energy (R=-0.72, P<0.005, ratings were averaged per participant, and across raters). That is, the greater reduction in perceived energy following sleep deprivation, the lonelier those sleep-deprived participants were rated by the judges (relative to their sleep rested state). Supporting an additional impact of perceived energy on social perception, greater perceived energy was associated with higher judges ratings of their desire to interact with that participant (R=0.650, P<0.01, ratings were averaged per participant, and across raters). These findings suggest that greater perceived energy attracts prosocial interactions and protects against evaluations of perceived loneliness by others.

Due to this combined contribution of loneliness and perceived energy on the desire to interact, the change in social interaction scores as a function of loneliness (mixed model 1) was compared to a model that includes both energy and loneliness as contributing to the desire to interact (mixed model 2). Three key findings emerged. (i) loneliness was a significant negative predictor of the desire to interact (β=-0.45±0.03, P<0.0001, mixed model 1), (ii) this negative effect of loneliness on the desire to interact remained significant even when controlling for energy ratings (β =-0.31±0.03, P<0.0001, mixed model 2), (iii) both models were significantly different from each other (χ^2=90.722, P<0.0001, likelihood ratio test). This demonstrates an additional contribution of loneliness to the desire to interact beyond the effects of perceived energy.

In summary, these findings reveal that both perceived energy levels as well as perceived loneliness are independent factors contributing to the choice of whether to socially interact with another individual, and are both similarly impacted by a lack of sleep.

Supplementary Figures

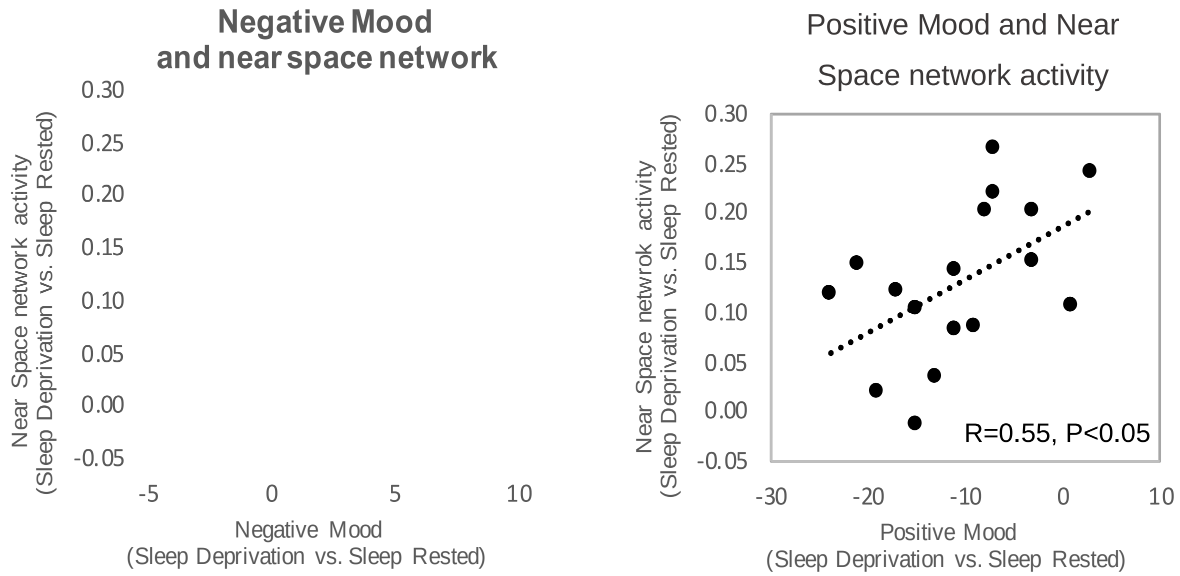

Supplementary Figure 1: Increased reactivity (human>object approach) within the Near Space network was associated with greater positive mood (right panel) and worse negative mood (left panel) following sleep deprivation. These associations are in the opposite direction to that observed for increased social withdrawal, and the overall main effect of sleep loss on brain activity within the Near Space network.

Supplementary References

1 Cacioppo, J. T. *et al.* Loneliness within a nomological net: An evolutionary perspective. *Journal of research in personality* **40**, 1054-1085 (2006).

2 Smith, S. S., Kozak, N. & Sullivan, K. A. An investigation of the relationship between subjective sleep quality, loneliness and mood in an Australian sample: can daily routine explain the links? *International Journal of Social Psychiatry* **58**, 166-171 (2012).

3 Cacioppo, J. T. *et al.* Do Lonely Days Invade the Nights? Potential Social Modulation of Sleep Efficiency. *Psychological Science* **13**, 384-387, doi:doi:10.1111/1467-9280.00469 (2002).

4 Jacobs, J. M., Cohen, A., Hammerman-Rozenberg, R. & Stessman, J. Global sleep satisfaction of older people: the Jerusalem Cohort Study. *Journal of the American Geriatrics Society* **54**, 325-329 (2006).

5 Mahon, N. E. Loneliness and sleep during adolescence. *Perceptual and motor skills* **78**, 227-231 (1994).

6 Cacioppo, J. T. *et al.* Loneliness and health: Potential mechanisms. *Psychosomatic Medicine* **64**, 407-417 (2002).

7 Hawkley, L. C., Preacher, K. J. & Cacioppo, J. T. Loneliness Impairs Daytime Functioning But Not Sleep Duration. *Health psychology : official journal of the Division of Health Psychology, American Psychological Association* **29**, 124-129, doi:10.1037/a0018646 (2010).

8 Pressman, S. D. *et al.* Loneliness, social network size, and immune response to influenza vaccination in college freshmen. *Health Psychology* **24**, 297 (2005).

9 Capitanio, J. P., Hawkley, L. C., Cole, S. W. & Cacioppo, J. T. A behavioral taxonomy of loneliness in humans and rhesus monkeys (Macaca mulatta). *PLoS One* **9**, e110307 (2014).

10 Adam, E. K., Hawkley, L. C., Kudielka, B. M. & Cacioppo, J. T. Day-to-day dynamics of experience–cortisol associations in a population-based sample of older adults. *Proceedings of the National Academy of Sciences* **103**, 17058-17063 (2006).

11 Cacioppo, J. T., Fowler, J. H. & Christakis, N. A. Alone in the crowd: The structure and spread of loneliness in a large social network. *Journal of personality and social psychology* **97**, 977 (2009).

12 Kurina, L. M. *et al.* Loneliness Is Associated with Sleep Fragmentation in a Communal Society. *Sleep* **34**, 1519-1526, doi:10.5665/sleep.1390 (2011).

13 Wilson, R. S., Krueger, K. R., Arnold, S. E. & et al. Loneliness and risk of alzheimer disease. *Archives of General Psychiatry* **64**, 234-240, doi:10.1001/archpsyc.64.2.234 (2007).

14 Holt, D. J. *et al.* Abnormalities in personal space and parietal–frontal function in schizophrenia. *NeuroImage: Clinical* **9**, 233-243 (2015).

15 Holt, D. J. *et al.* Neural correlates of personal space intrusion. *Journal of Neuroscience* **34**, 4123-4134 (2014).

16 Teneggi, C., Canzoneri, E., di Pellegrino, G. & Serino, A. Social Modulation of Peripersonal Space Boundaries. *Current Biology* **23**, 406-411, doi:https://doi.org/10.1016/j.cub.2013.01.043 (2013).

17 Makin, T. R., Holmes, N. P. & Zohary, E. Is that near my hand? Multisensory representation of peripersonal space in human intraparietal sulcus. *Journal of Neuroscience* **27**, 731-740 (2007).
18 Bremmer, F. *et al.* Polymodal motion processing in posterior parietal and premotor cortex: a human fMRI study strongly implies equivalencies between humans and monkeys. *Neuron* **29**, 287-296 (2001).
19 Heed, T., Habets, B., Sebanz, N. & Knoblich, G. Others' actions reduce crossmodal integration in peripersonal space. *Current Biology* **20**, 1345-1349 (2010).
